# Sedentary behaviour, physical activity, and sleep among office workers during the COVID-19 pandemic: a comparison of Brazil and Sweden

**DOI:** 10.1186/s12889-022-14666-9

**Published:** 2022-11-28

**Authors:** Luiz Augusto Brusaca, Leticia Bergamin Januario, Svend Erik Mathiassen, Dechristian França Barbieri, Rafaela Veiga Oliveira, Marina Heiden, Ana Beatriz Oliveira, David M. Hallman

**Affiliations:** 1 Laboratory of Clinical and Occupational Kinesiology, Department of Physical Therapy, Federal University of São Carlos, Washington Luiz Road, km 235, SP310, 13565-905, São Carlos, São Paulo, Brazil; 2 Centre for Musculoskeletal Research, Department of Occupational Health Sciences and Psychology, University of Gävle, SE-801 76 Gävle, Sweden; 3 Department of Industrial Engineering, Clemson University, 277A Freeman Hall, SC, 29634 Clemson, USA

**Keywords:** Occupational health, Public health, Accelerometry, Compositional data analysis, 24-hour movement behaviour, Physical inactivity

## Abstract

**Background:**

The COVID-19 pandemic has affected the physical behaviours of office workers worldwide, but studies comparing physical behaviours between countries with similar restrictions policies are rare. This study aimed to document and compare the 24-hour time-use compositions of physical behaviours among Brazilian and Swedish office workers on working and non-working days during the pandemic.

**Methods:**

Physical behaviours were monitored over 7 days using thigh-worn accelerometers in 73 Brazilian and 202 Swedish workers. Daily time-use compositions were exhaustively described in terms of sedentary behaviour (SED) in short (< 30 min) and long (≥30 min) bouts, light physical activity (LPA), moderate-to-vigorous physical activity (MVPA), and time-in-bed. We examined differences between countries using MANOVA on data processed according to compositional data analysis. As Swedish workers had the possibility to do hybrid work, we conducted a set of sensitivity analyses including only data from days when Swedish workers worked from home.

**Results:**

During working days, Brazilian office workers spent more time SED in short (294 min) and long (478 min) bouts and less time in LPA (156 min) and MVPA (50 min) than Swedish workers (274, 367, 256 and 85 min, respectively). Time spent in bed was similar in both groups. Similar differences between Brazilians and Swedes were observed on non-working days, while workers were, in general, less sedentary, more active and spent more time-in-bed than during working days. The MANOVA showed that Brazilians and Swedes differed significantly in behaviours during working (*p* <  0.001, η_p_^2^ = 0.36) and non-working days (*p* <  0.001, η_p_^2^ = 0.20). Brazilian workers spent significantly more time in SED relative to being active, less time in short relative to long bouts in SED, and more time in LPA relative to MVPA, both during workdays and non-workdays. Sensitivity analyses only on data from days when participants worked from home showed similar results.

**Conclusions:**

During the COVID-19 pandemic Brazilian office workers were more sedentary and less active than Swedish workers, both during working and non-working days. Whether this relates to the perception or interpretation of restrictions being different or to differences present even before the pandemic is not clear, and we encourage further research to resolve this important issue.

**Supplementary Information:**

The online version contains supplementary material available at 10.1186/s12889-022-14666-9.

## Introduction

Since the coronavirus disease (COVID-19) outbreak in 2019, a considerable literature addressing effects of the pandemic on physical behaviours has appeared [1–4]. Most of these studies have compiled information from a general population facing the challenges of shifting to working either from home (WFH) or in a hybrid set-up, i.e., working with a mix of days WFH and working at the office (WAO). The main findings in these reviews [1–4] are similar to those in an online survey study by Trabelsi et al. [5], showing for a large international population that daily time spent in physical activity (PA) decreased and sitting time increased during the COVID-19 pandemic. Similarly, a review by Ráthonyi et al. [6] showed that physical behaviours of office workers were affected by restrictions during the pandemic in the direction of decreased PA, increased sedentary behaviour (SED) and increased sleeping time.

Even before the pandemic, office workers spent most of their day in SED, both during working and non-working hours, and they moved for less than 10% (i.e., about 100 min) of the day [7, 8]. High levels of SED and low levels of PA have been associated with detrimental health effects, including increased risks of developing chronic non-communicable diseases (e.g., type 2 diabetes, overweight, obesity, metabolic syndrome, cancer and cardiovascular diseases) [9–11]. Some studies have shown that the harmful effects on health of extensive time spent in SED can be counteracted or mitigated if more time is spent in vigorous PA [11, 12]. In addition, a sleep duration of 7 to 8 hours per night is positively associated with health outcomes, and both too much and too little sleep may lead to health problems [13]. However, daily behaviours are inherently co-dependent and constrained because they share time within a finite 24-hour window. More time can be spent in one behaviour only at the cost of reducing time on one or more other behaviours, so that the fixed total of 24 hours, or 100%, is maintained [14, 15].

The changes in physical behaviours during the COVID-19 pandemic likely differed between countries due to different social restrictions adopted in order to slow down the transmission of the virus [16, 17]. Thus, the severity of restrictions has varied between countries, for instance regarding the distance people could travel from their homes, and the allowances for outdoor activity [18]. In contrast to many other countries implementing quite restricted and mandatory measures, the authorities in Brazil and Sweden largely relied on recommendations and voluntary measures, as implemented through individual responsibility. Thus, during the pandemic, many Swedish workers had a formal opportunity to choose WFH or WAO, even if WFH was recommended [19].

Brazil and Sweden thus appear similar in the extent of restrictions. The two countries do, however, differ in terms of socioeconomic status, culture, work environment, and the extent of physical inactivity [20, 21]. Prior to the COVID-19 pandemic, WFH was not common in Brazil (prevalence of 1 to 5%; International Labour Organization [22]) and the only legislation present was related to teleworking (article 75 of the law n° 13.467 of 2017), which does not deal with WFH. In contrast, remote work was already common in Sweden (prevalence of 20%) and it was regulated before the pandemic [23]. Thus, Brazilian office workers appeared to have fewer opportunities for choosing where to work during the pandemic (i.e., choose between WFH and WAO) compared to Swedes [19, 24]. Brazilians in general also have lower socioeconomic status, and are more sedentary and less physically active than Swedes [20, 21]. Therefore, comparing effects of the pandemic on physical behaviours of Brazilian and Swedish office workers would shed light on the extent to which behaviours were different in countries with the same level of restrictions but different demographic and social characteristics.

Most studies investigating the physical behaviour of office workers during the COVID-19 pandemic have been based on self-reported measures [1, 2, 6]. However, studies have shown that self-reported data may suffer from bias [25, 26], and that the extent of bias may be related to the status of the respondents [27, 28]. To come around the limitations associated with self-reports, data on time spent in different physical behaviours need to be assessed using wearable sensors, such as accelerometers, allowing accurate assessment of physical behaviours around the clock for several days [29, 30].

A 24-hour objective assessment of time-use allows a comprehensive understanding of physical behaviours in occupational as well as non-occupational contexts, and it also provides a basis for understanding of how behaviours interact, and how they together may influence health outcomes [15, 31]. Additionally, evidence of 24-hour behaviours allows for the formulation of valid guidelines for physical behaviours [31, 32], which can effectively support strategies to prevent sedentary lifestyles and promote better health. The importance of addressing behaviours in a 24-hour perspective is also reflected in recent guidelines, such as The Canadian 24-hour movement guidelines for adults [33].

To our knowledge, detailed data are lacking on 24-hour time-use compositions of SED, PA and sleep of office workers from different countries during the pandemic, measured using accelerometers. Understanding 24-hour time-use compositions of physical behaviours during the pandemic in countries with less severe social restrictions, while acknowledging differences in demography and socioeconomics, may assist future post-pandemic recommendations, including guidelines for WFH. It may even contribute to a better preparedness in the event of a future pandemic scenario. Therefore, the aim of this study was to document and compare 24-hour time-use compositions of sedentary behaviour, physical activity and time-in-bed (as a proxy for sleep) among Brazilian and Swedish office workers at working and non-working days during the COVID-19 pandemic, on the basis of recordings by wearable sensors.

## Methods

### Design and study population

This cross-sectional study was conducted using data from two studies, one in Brazil and one in Sweden. The Brazilian study was designed specifically to investigate behaviours among office workers in public and private organizations WFH during the COVID-19 pandemic. The Swedish study uses data from an ongoing cohort evaluating flexible work in different public and private organizations (Flexible work: Opportunity and Challenge – FLOC) [34]. In Brazil, data were collected between September 2020 and April 2021, and in Sweden between June 2020 and June 2021.

**Fig. 1:**
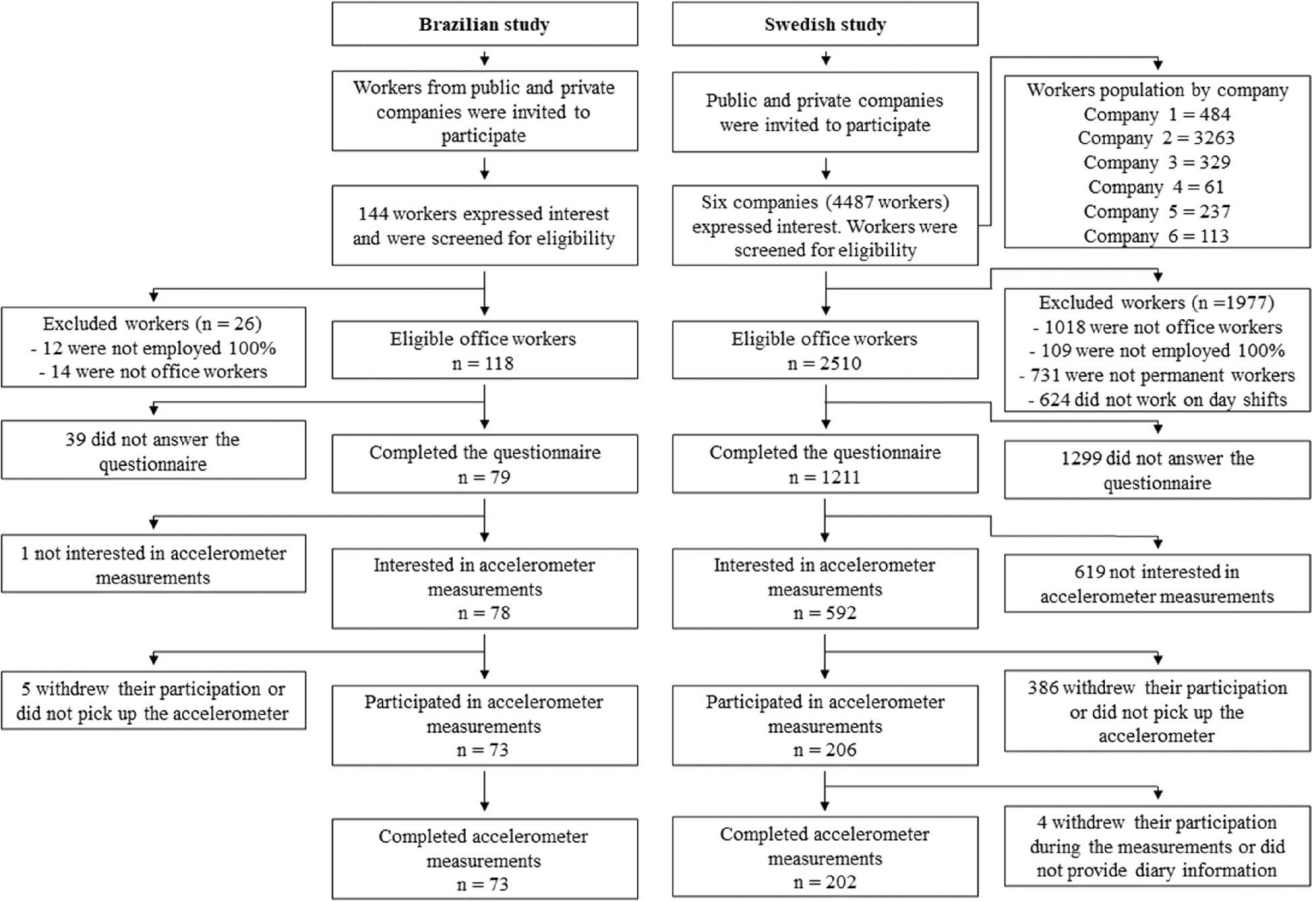
Flow chart of participant recruitment and data collection. Note: The number of “Excluded workers” in different categories in the Swedish study exceeds the total number (*n* = 1977), because a participant could be excluded based on more than one criterion

For this study, we included white-collar workers who were predominantly involved in office-based tasks (e.g., answering emails, data entry, processing documents, and browsing the internet) with a permanent full-time employment contract. In Brazil, workers were invited to participate through advertisements published through the social media of the regional university. In Sweden, several companies in different regions of the country were invited to participate in the cohort. If the manager of a certain company decided to participate, a formal presentation of the project was given to the whole company. The manager was responsible for providing information about all employees at the company, including number of hours worked, type of employment and professional email. Each worker in a particular company was invited to participate in the study via e-mail. The recruitment process for both countries is illustrated in Fig. 1.

The studies were conducted in accordance with the Helsinki declaration, and all participants provided their written informed consent prior to entering the studies. The Brazilian study was approved by the Human Ethics Committee of the Federal University of São Carlos (São Carlos, SP, Brazil; registration process #38136420.9.0000.5504) and the Swedish study by the Swedish Ethical Review Authority (decision 2019–06220).

### Data collection

In general, data collection by questionnaires and measurements using wearable sensors were similar in both studies. All workers meeting the inclusion criteria were asked to complete a web-based questionnaire containing demographic/personal information, including questions that were similar in Brazil and Sweden, i.e. gender, age, company position (manager or employee), work schedule (business hours or varying working hours [shifting between business hours, evenings, and weekends]), type of contract (permanent contract, temporary employment or fixed-term contracts), education (secondary or higher education), and smoking status (yes or no). Respondents were also asked if they were interested in participating in measurements of physical behaviours. At a positive answer, the potential participant, both in Brazil and in Sweden, was contacted via e-mail or a messaging application (WhatsApp - Meta, Inc.) and a meeting was arranged between the researcher and the participant. During this meeting, which lasted about 30 min, a member of the research team fixed the accelerometer on the worker’s right thigh and measured body weight and height. All feasible biosafety precautions were taken as needed because of the COVID-19 pandemic, and in some cases, conditional on the participant’s request, we had the participant attach the devices remotely. The participants then received the sensors in a sealed container and instructions for attaching the accelerometer were communicated through a pamphlet and a video, pre-recorded by the researchers. All participants involved in the remote session were contacted by the researchers in a video call, to check if they had experienced any potential issues during the procedure. In total, 3 (4%) Brazilian participants and 33 (16%) Swedish participants attached the devices using this remote procedure.

### Assessment of physical behaviours

Physical behaviours were monitored over 7 days, in Brazil using the ActivPAL Micro 4 accelerometer (PAL technologies, Glasgow, Scotland) sampled at 20 Hz, and in Sweden with the Axivity AX3 (Axivity, Newcastle, UK) sampled at 25 Hz. The sensors were fixed with double-sided adhesive tape on the front of the worker’s right thigh, midway between the hip and the knee joint. Accelerometer data were downloaded using the manufacturers’ software (PAL Software Suite Version 8 in Brazil, and OMGUI Version 1.0.0.43 in Sweden) and then the data were processed using a custom-made MATLAB program, *ActiPASS* [29, 30, 35]. The *ActiPASS* program determines the time spent in each of an exhaustive array of physical behaviours with a confirmed good validity [29, 30, 35], and provides similar results for data collected using ActivPAL and Axivity devices [36]. During the measurement period, participants recorded in a diary whether the day was a non-working day or a working day, and noted if they worked from home or at the office, and they also noted time-in-bed; i.e., the time they went to bed in the evening and the time they woke up. Only days with complete 24-hour measurements were included for further analyses. Also, the working day had to contain at least 4 hours of work to be included in the analyses [37–39].

We categorized time spent in different physical behaviours over a 24-hour day in four categories: sedentary (SED: sitting and lying), light physical activity (LPA: standing, moving [i.e., dynamic standing] and slow walking [< 100 steps/min]), moderate-to-vigorous physical activity (MVPA: fast walking [> 100 steps/min], walking stairs, running and cycling) and time-in-bed (TIB); as done previously [24, 40]. The first three were based on the *ActiPASS* results, and the last was identified on basis of the diary. Additionally, in order to measure the temporal pattern of behaviour (i.e., the variation; Mathiassen [41]), we categorized time spent in SED in short bouts, < 30 min, and long bouts, ≥30 min [7].

### Time-use compositions

#### Descriptive parameters

We processed the 24-hour time-use compositions according to compositional data analysis (CoDA) procedures [14, 15, 42] using the package ‘compositions’ v2.0-2 [43] in R v4.2.0 [44].

Daily time spent in each behaviour was averaged over all measured working days and over all non-working days for each worker. Then, for each behaviour during working and non-working days, the data were expressed in terms of compositional means, in minutes (closed to a total duration of 1440 min, i.e., 24-hour) as well as percentages (closed to 100%). Differences in each behaviour between countries were expressed in terms of a log-transformed ratio between the geometric means of the Brazilian group (numerator) and Swedish group (denominator). A positive value of the log-ratio indicates that workers from Brazil spent more time in that behaviour than workers from Sweden, and vice versa if the value is negative. The log-ratio was expressed in absolute terms with 95% confidence intervals based on bootstraps of 1000 virtual ratios drawn from the behaviours observed in each country, and as a percentage difference using the following formula: 100 – (100 * exp.(log-ratio of geometric means)) [45, 46].

#### Isometric log-ratio (ilr) transformations

Following CoDA procedures, the 24-hour time-use compositions of physical behaviours of working and non-working days were transformed into sets of four isometric log-ratio (ilr) coordinates, using a sequential binary partition [42]. This set of coordinates describes ratios of behaviours tailored to our research question, and specifically reflect contrasts in behaviour that we wished to address. The ilr-coordinates were defined as follows:

$${\textrm{ilr}}_1=\sqrt{\frac{4}{5}}\ln \left(\frac{\textrm{TIB}}{\sqrt[4]{\textrm{SED}\ \textrm{in}\ \textrm{bouts}<30\ \min \ast \textrm{SED}\ \textrm{in}\ \textrm{bouts}\ge 30\ \min \ast \textrm{LPA}\ast \textrm{MVPA}}}\right)$$

$${\textrm{ilr}}_2=\ln \left(\frac{\sqrt{\textrm{SED}\ \textrm{in}\ \textrm{bouts}<30\ \min \ast \textrm{SED}\ \textrm{in}\ \textrm{bouts}\ge 30\ \min }}{\sqrt{\textrm{LPA}\ast \textrm{MVPA}}}\right)$$

$${\textrm{ilr}}_3=\sqrt{\frac{1}{2}}\ln \left(\frac{\textrm{SED}\ \textrm{in}\ \textrm{bouts}<30\ \min }{\textrm{SED}\ \textrm{in}\ \textrm{bouts}\ge 30\ \min}\right)$$

$${\textrm{ilr}}_4=\sqrt{\frac{1}{2}}\ln \left(\frac{LPA}{\textrm{MVPA}}\right)$$

ilr_1_ expresses the ratio of time-in-bed to time spent awake (i.e., all other behaviours); ilr_2_ expresses time spent sedentary (both short bouts and long bouts) relative to non-sedentary behaviours (non-SED; i.e., light and moderate-to-vigorous physical activity); ilr_3_ expresses time spent sedentary in short bouts relative to long bouts; and ilr_4_ expresses time spent in light physical activity relative to moderate-to-vigorous physical activity. The transformation of compositional data into a set of ilr-coordinates allows data to be analysed further using standard statistical methods [14, 15].

### Statistical analysis

#### Descriptive statistics

Characteristics of the study sample were described using frequencies and percentages for categorical data and means and standard deviation (SD) for continuous variables. Physical behaviours during working and non-working days were illustrated using cumulative distribution plots in the standard, non-transformed space, and also by the differences in behaviour between countries expressed in terms of log-ratios of geometric means of behaviours with bootstrap confidence intervals [45, 46]. Thus, the log-ratio metric was used as a descriptive variable, complementary to the main analysis described below.

#### Data analysis

The ilr-transformed data were analysed in an unadjusted model using one-way multivariate analysis of variance (MANOVA) to assess the difference between Brazil and Sweden in physical behaviours during working and non-working days, and then in an adjusted model using one-way multivariate analysis of covariance (MANCOVA) controlling for sex, age, company position, education and body mass index (BMI). In all analyses, partial eta squared (η_p_^2^) was used as a measure of effect size, and the corresponding *p*-value as a complementary metric for evaluating statistical significance. Following the results of the unadjusted model, univariate post-hoc tests of pairwise differences were applied, using Cohen’s *d* as a measure of effect size, and *p*-values as measures of statistical significance.

#### Sensitivity analysis

In the main analysis (described above), we included all working days of the entire sample in Brazil and Sweden. Since the Brazilian group was composed only of workers who were WFH and the Swedish workers had the possibility to do hybrid work, we conducted a set of sensitivity analyses, using the same procedure as described above, comparing the Brazilian group only with data from Swedish WFH days.

## Results

### Flow of participants

Of a total of 144 Brazilian workers who expressed interest in participating in the study, 118 met the inclusion criteria (Fig. 1). Of these 118 office workers, 79 completed the questionnaire (response rate 67%) and 73 took part in the accelerometer measurements. In Sweden, six companies participated, including a total of 4487 workers, of which 2510 met the inclusion criteria, and 1211 responded to the questionnaire (response rate 48%). Of the 1211 office workers, 592 were interested in the accelerometer measurements and 202 completed them (Fig. 1).

### Characteristics of the study population

In general, descriptive statistics for the entire sample showed that the Brazilian (*n* = 73) and Swedish (*n* = 202) groups were composed of slightly more female than male participants (53% of Brazilians and 56% of Swedes). Mean age of the Brazilian and Swedish workers was 33.1 (SD 9.1) and 42.8 (SD 10.4) years, respectively. Only 7% of Brazilian workers had a management position in the company, while 16% of the Swedish group were managers. On the other hand, more Brazilians (93%) had higher education compared to Swedes (83%). The percentage of smokers was similar in both groups (4% of Brazilians and 3% of Swedes). Brazilian workers had a mean BMI of 26.8 (SD 4.8) kg/m^2^ and Swedish workers 25.2 (SD 3.8) kg/m^2^.

The description of the accelerometer data showed that the 73 workers from Brazil and 202 workers from Sweden were recorded for a total of 10,029 hours (504 days) and 31,917 hours (1536 days), respectively, with, on average, 137.4 hours (SD 18.6) and 158.0 hours (SD 29.4) of data per worker. Of all data collected, 145 days from the Brazilian group and 441 from the Swedish were excluded for having less than 24 hours of measurement, and another 3 days (Brazil) and 11 days (Sweden) for not having at least 4 hours of work. This left 356 and 1084 full days for further analysis, with each worker being measured, on average, for 4.9 days (SD 0.7; range 1-6) and 5.4 days (SD 1.2; range 1-7), respectively.

Table 1 presents the resulting data in terms of workers with valid working and non-workings days of measurement. In general, the sociodemographic characteristics of workers with valid days of measurement were similar to the entire sample described above.

**Table 1:** Demographic and social characteristics of participants with accelerometry measurements in Brazil and Sweden; as well as information from the accelerometer data

	Brazil		Sweden	
	Working days *n* = 72	Non-working days *n* = 70	Working days *n* = 199	Non-working days *n* = 184
Sex ^a^				
Female	38 (52.8)	36 (51.4)	111 (55.8)	105 (57.1)
Male	34 (47.2)	34 (48.6)	88 (44.2)	79 (42.9)
Age (years) ^a^	33.2 (9.2)	33.4 (9.1)	42.8 (10.4)	42.8 (10.5)
Company position ^a^				
Manager	5 (6.9)	5 (7.1)	32 (16.1)	23 (12.5)
Employee	67 (93.1)	65 (92.9)	167 (83.9)	161 (87.5)
Education ^a^				
Secondary education	5 (6.9)	5 (7.1)	34 (17.1)	30 (16.3)
Higher education	67 (93.1)	65 (92.9)	165 (82.9)	154 (83.7)
Smokers (yes) ^a^	3 (4.2)	3 (4.3)	5 (2.5)	5 (2.7)
Body mass index (kg/m^2^) ^b^	26.8 (4.8)	26.6 (4.8)	25.1 (3.8)	25.3 (3.9)
Accelerometer data				
Total hours (days) recorded	4968 (207)	3576 (149)	16,873 (703)	9144 (381)
Mean hours recorded	69.0 (12.0)	51.1 (12.9)	84.8 (20.7)	49.4 (15.0)
Mean days recorded	2.9 (0.5; range 1-4)	2.1 (0.5; range 1-5)	3.5 (0.9; range 1-5)	2.1 (0.6; range 1-5)

Results are presented as mean (standard deviation between subjects) and number of workers (percentage)

^a^Self-reported information from online questionnaire

^b^Objectively measured

### Physical behaviours expressed in standard space

**Fig. 2:**
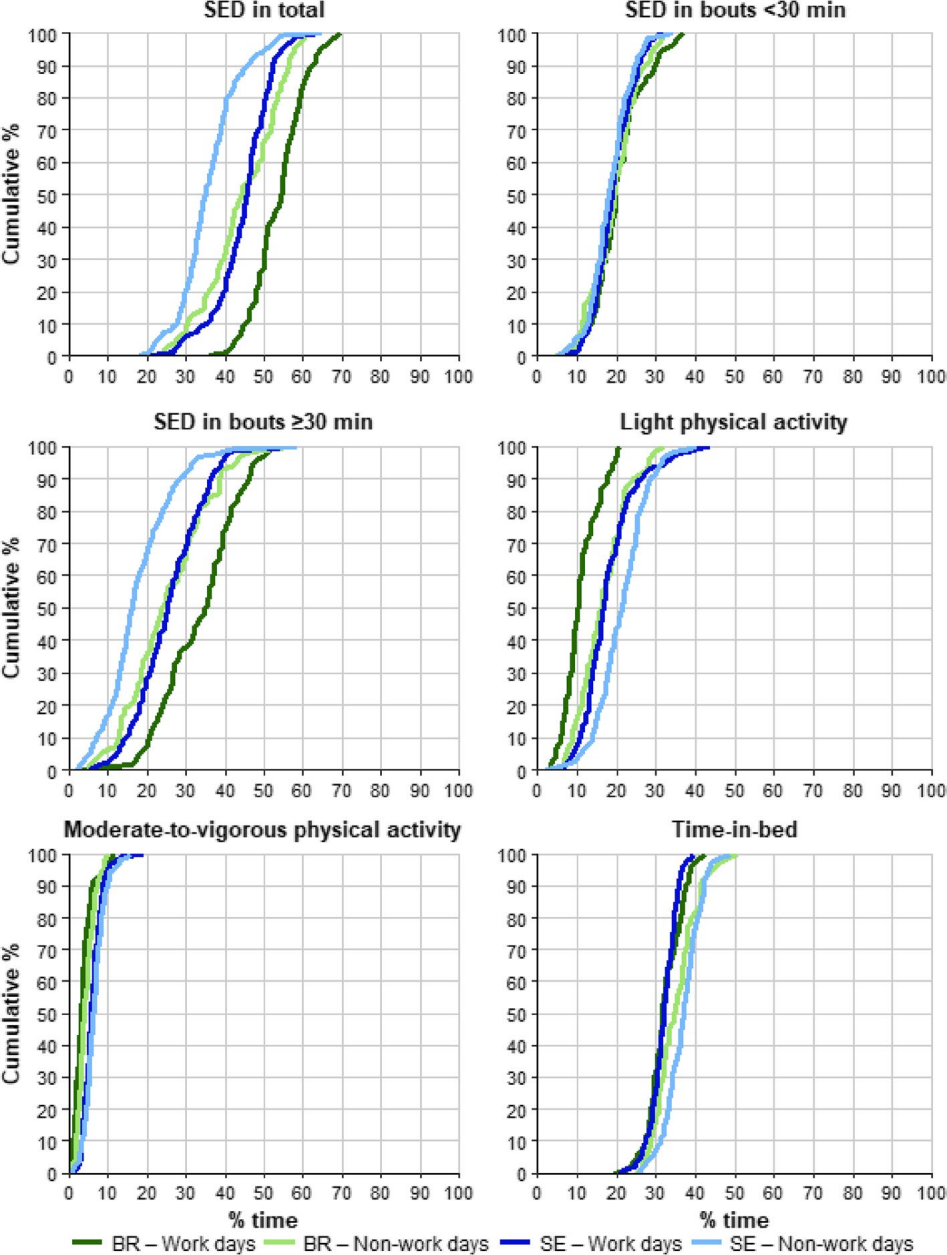
Cumulative distributions of percentages of time spent sedentary (SED) in total, SED in bouts < 30 min, SED in bouts ≥30 min, light physical activity, moderate-to-vigorous physical activity, and in bed. Distributions are shown for Brazilian (BR; green) and Swedish (SE; blue) office workers during working days (dark tones) and non-working days (light tones)

Cumulative distributions of SED in total, SED in bouts < 30 min, SED in bouts ≥30 min, LPA, MVPA and TIB during working and non-working days are illustrated in Fig. 2. During working days, 73% of the Brazilian workers and 25% of the Swedish workers were SED for more than 50% of the 24-hour day (Fig. 2). Within total SED time, a larger proportion was accumulated in long bouts ≥30 min than in short bouts < 30 min (Fig. 2). Swedish workers accumulated more time in LPA than Brazilian workers, and time spent in MVPA was accumulated only to a minor extent in both groups (Fig. 2). Time-in-bed occurred for more than 30% of the day for 68% of Brazilian workers and 74% of the Swedes (Fig. 2). On non-working days, the proportions of Brazilian and Swedish workers accumulating 50% of their day in SED were smaller (36% of Brazilians and 7% of Swedes) compared to working days; SED in long bouts was still the dominant SED behaviour, at least for the Brazilian workers (Fig. 2). Workers from both groups accumulated more time in LPA and MVPA during non-working days than during working days. The proportion of Brazilian and Swedish workers accumulating more than 30% of their day in bed was 84 and 93%, respectively, during non-working days.

### Physical behaviours expressed as compositions

The compositional mean of time spent in different physical behaviours on all working days, working days WFH, and non-working days are shown in Table 2. In general, when expressing data as compositional mean values, the distributions of behaviours were similar to those in standard space illustrated in Fig. 2.

**Table 2:** Compositional mean (SD between participants) in minutes per day and percentage of time of each behaviour of Brazilian and Swedish office workers; for working days, working days only working from home (WFH), and non-working days

	Brazil		Sweden	
	Minutes	% Time	Minutes	% Time
*Working days*	*n* = 72		*n* = 199	
SED in short bouts <30 min	294 (92)	20.4 (6.4)	274 (71)	19.0 (4.9)
SED in long bouts ≥30 min	478 (144)	33.2 (10.0)	367 (124)	25.5 (8.6)
Light PA	156 (63)	10.8 (4.4)	256 (97)	17.8 (6.7)
Moderate-to-vigorous PA	50 (34)	3.5 (2.3)	85 (35)	5.9 (2.4)
Time-in-bed	462 (63)	32.1 (4.4)	458 (49)	31.8 (3.4)
*Working days only WFH*	*n* = 72		*n* = 134	
SED in short bouts <30 min	294 (92)	20.4 (6.4)	272 (84)	18.9 (5.8)
SED in long bouts ≥30 min	478 (144)	33.2 (10.0)	374 (133)	25.9 (9.2)
Light PA	156 (63)	10.8 (4.4)	245 (102)	17.0 (7.1)
Moderate-to-vigorous PA	50 (34)	3.5 (2.3)	81 (35)	5.6 (2.4)
Time-in-bed	462 (63)	32.1 (4.4)	469 (51)	32.6 (3.5)
*Non-working days*	*n* = 70		*n* = 184	
SED in short bouts <30 min	279 (86)	19.4 (6.0)	263 (74)	18.2 (5.1)
SED in long bouts ≥30 min	359 (155)	25 (10.8)	251 (124)	17.4 (8.6)
Light PA	237 (90)	16.4 (6.2)	305 (92)	21.2 (6.4)
Moderate-to-vigorous PA	61 (32)	4.3 (2.2)	93 (39)	6.4 (2.7)
Time-in-bed	504 (78)	35.0 (5.4)	529 (64)	36.7 (4.5)

*SED* sedentary behaviour, *PA* physical activity

**Fig. 3:**
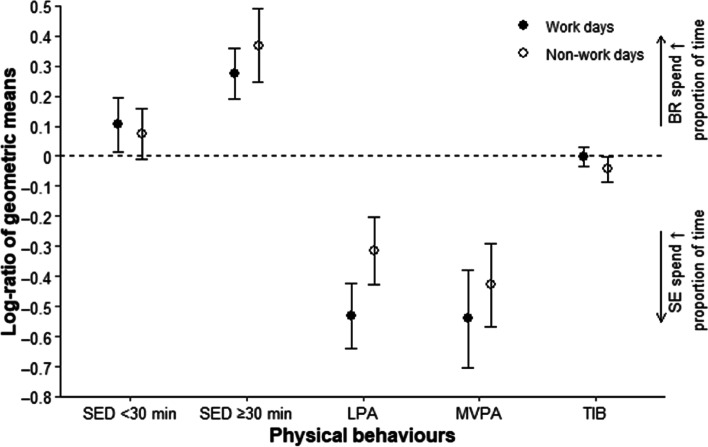
Log-ratio (circles) with bootstrap 95% percentile confidence intervals (vertical lines) of the geometric means of the Brazilian group (numerator) and Swedish group (denominator) at working (filled circles) and non-working days (unfilled circles). A positive log-ratio shows that Brazilian office workers spent more time in that behaviour compared to the Swedish office workers, and vice versa if the log-ratio is negative. If the confidence interval includes zero, the difference was not significant at *p* <  0.05. Abbreviations: SED, sedentary behaviour; LPA, light physical activity; MVPA, moderate-to-vigorous physical activity; TIB, time-in-bed

Figure 3 shows the estimated log-ratios of the geometric means of each physical behaviour during working and non-working days in the Brazilian and Swedish groups, and the associated 95% bootstrap percentile confidence intervals. During working days, the percentage time spent by Brazilian workers in SED in bouts < 30 min, SED in bouts ≥30 min and in bed was 11.0% (log-ratio of geometric means 0.10), 31.4% (0.27) and 0.1% (0.001) larger than among Swedish workers, while time in LPA and MVPA were 41.1% (− 0.53) and 41.7% (− 0.54) less. On non-working days, the general difference in behaviour patterns between Brazilians and Swedes were similar to that during working days (Fig. 3).

### Statistical analysis of physical behaviours expressed as compositions

The one-way MANOVA (i.e., unadjusted model) of the set of the four ilr’s as a whole suggested a significant difference between the Brazilian and Swedish groups in behaviours during working days (F(4, 266) = 37.35, *p* <  0.001, *Λ* = 0.64, η_p_^2^ = 0.36). The one-way MANCOVA (i.e., adjusted model) confirmed that the groups differed in their overall 24-hour time-use composition even after controlling for sex, age, company position, education and BMI (F(4, 261) = 39.25, *p* <  0.001, *Λ* = 0.62, η_p_^2^ = 0.38). Sex, age and education were the only significant covariates (*p* <  0.5) in the adjusted model (full adjusted model: see Supplementary Table 1 in Additional file 1). On non-working days, the Brazilian and Swedish groups differed in their overall 24-hour behaviour (F(4, 249) = 15.32, *p* < 0.001, *Λ* = 0.80, η_p_^2^ = 0.20); confirmed in the adjusted model (F(4, 244) = 17.40, *p* < 0.001, *Λ* = 0.78, η_p_^2^ = 0.22). The significant covariates (*p* < 0.5) in the adjusted model were sex, age and BMI (full adjusted model: see Supplementary Table 1 in Additional file 1).

Results of the univariate post-hoc tests of the ilr coordinates are shown in Table 3. A positive ilr shows that the time spent in the numerator behaviour was larger than that in the denominator, and an ilr having a negative value shows the opposite. Thus, the univariate test for working days showed that the ratio of TIB to time spent awake (ilr_1_); SED in short and long bouts to non-SED, i.e., LPA an MVPA (ilr_2_); and LPA to MVPA (ilr_4_) was larger in the Brazilian group than in the Swedish. Time spent in short bouts of SED relative to long bouts (ilr_3_) was smaller in the Brazilian group than in the Swedish. Behaviours during non-working days showed the same results as working days, except that the difference in ilr_1_ was now insignificant (Table 3).

**Table 3:** Mean ilr coordinates of each group; results of the univariate post-hoc tests

	Brazil	Sweden	*t*	MD [95% CI]	*p*	*d*
*Working days*						
ilr_1_: TIB/awake	0.92	0.72	5.49	0.20 [0.13; 0.27]	**< 0.001**	0.82
ilr_2_: SED/non-SED	1.54	0.79	9.94	0.76 [0.61; 0.91]	**< 0.001**	1.45
ilr_3_: SEDshort/SEDlong	−0.33	−0.18	−2.31	−0.15 [−0.27; −0.02]	**0.02**	0.33
ilr_4_: LPA/MVPA	0.90	0.79	2.10	0.11 [0.01; 0.22]	**0.04**	0.30
*Non-working days*						
ilr_1_: TIB/awake	0.92	0.90	0.61	0.02 [−0.05; 0.09]	0.54	0.09
ilr_2_: SED/non-SED	1.00	0.41	7.21	0.59 [0.43; 0.75]	**< 0.001**	1.02
ilr_3_: SEDshort/SEDlong	−0.14	0.10	−3.29	−0.24 [−0.38; −0.10]	**0.001**	0.46
ilr_4_: LPA/MVPA	1.00	0.87	2.63	0.14 [0.03; 0.24]	**0.01**	0.38

*ilr* isometric log-ratio, *t t*-test statistic, *MD* mean difference between Brazilian and Swedish group, *95% CI* lower and upper limit of a 95% confidence interval on the mean difference, *p* significance level, *d* Cohen’s effect size *d*, *TIB* time-in-bed, *SED* sedentary behaviour, *non-SED* non-sedentary behaviour, *LPA* light physical activity, *MVPA* moderate-to-vigorous physical activity, Results with *p* < 0.05 are shown in bold

### Sensitivity analyses

When excluding 353 WAO days from Swedish workers and only analysing WFH days, the descriptive and statistical results were similar to the main analysis, with a few minor exceptions (see Supplementary Figs. 1 and 2, and Tables 2 and 3 in Additional file 1).

## Discussion

This study documented, during working and non-working days, the 24-hour time-use compositions of sedentary behaviour, physical activity and time-in-bed (i.e., proxy for sleep) of Brazilian and Swedish office workers during the COVID-19 pandemic and also examined to which extent these compositions differed between countries. To date, no other study has compared the 24-hour time-use compositions of office workers measured using wearable sensors (i.e., accelerometers) between countries with similar restriction policies, but with acknowledged differences in demographic and socioeconomics. Therefore, examining the compositions of time spent during work for these workers is important to support post-pandemic interventions and recommendations, including guidelines for WFH. Likely a substantial proportion of workers will continue to work from home or in a hybrid model (i.e., both WFH and WAO) after the pandemic [47].

Compared with Swedes, Brazilian workers spend more TIB relative to time awake on working days, more time in SED (both short bouts and long bouts) relative to in non-SED, more time SED in long bouts relative to short bouts, and more time in LPA relative to MVPA (Table 3). Similar results were found during non-working days, the only exception being that the relative proportions of TIB were similar for workers in both countries (Table 3). Sensitivity analysis of data only from working days spent at home showed similar results to the analysis of working days in general, however with a slightly smaller effect size. The results indicate that differences between countries may be explained only to a minor extent by the opportunity in Sweden to work at the office [19], and that other factors also contributed to the differences between the physical behaviours of Brazilian and Swedish workers found in this study [20, 21].

Previous studies of work during the pandemic corroborate our findings, in reporting that office workers spend extensive amount of time sedentary during working and non-working days [6, 24] irrespective of the workplace, i.e. whether it is at home or at the office [19, 48]. Even before the COVID-19 pandemic, extensive sedentariness was an issue among office workers [8, 49]. The literature indicates that SED has increased during the pandemic compared to before [1–4, 6], which may have been an effect of physical distancing and isolation imposed by the pandemic. Both distancing and isolation, to an extent depending on the country and when the pandemic occurred [18], diminished or eliminated the autonomy of people to leave their homes and engage in regular activities (e.g., school, work, fitness training), as well as utilize community resources (e.g., parks, playgrounds, walking trails). As a result, strategies to contain the virus may have increased the prevalence of physical inactivity worldwide, reducing the already low levels of physical activity in some occupational groups [21, 50]. The detrimental effect of the pandemic may have been larger in low- and middle-income countries, such as Brazil, compared with high-income countries, such as Sweden [20, 50]. Since, however, Brazilian office workers do not have a very low income, it appears that additional factors may have contributed to the difference in physical behaviours between Brazil and Sweden. This could, for example, be a low awareness among the Brazilian workers of the benefits of physical activity for health and well-being [51]. Therefore, this indicates that Brazilian office workers may be in particular need of preventive actions to reduce sedentary behaviour and increase physical activity.

Physical activity, sedentary behaviours and sleep have been investigated independent of one another for a long time, but recent research highlights the importance of addressing behaviours in a 24-hour perspective [14, 15, 32], as it provides a better understanding of how behaviours interact and how they may influence health outcomes together [31]. The importance of addressing behaviours in a 24-hour perspective is also reflected in the Canadian 24-hour movement guidelines [33]. This guideline for adults aged 18–64 years recommends at the most 8 sedentary hours per day, and recommends breaking up sitting as often as possible, as well as spending at least 150 min per week in moderate-to-vigorous physical activity, along with several hours of light activity and/or standing, and 7 to 9 hours per night of good-quality sleep [33]. In general, the population samples in our study did not achieve a balanced 24-hour time-use according to the Canadian guidelines. When examining the behaviours of each individual participant during working days, none of the Brazilian workers and only 13 Swedish workers achieved a 24-hour time-use that fulfilled the Canadian guidelines. On non-working days, four Brazilian workers and 34 Swedish workers met the guidelines. This may deserve particular attention in policy planning [31, 33]. When checking each component of the guideline, the recommendation to limit SED to 8 hours or less per day was the least likely to be achieved by most workers in our sample. A majority of Brazilian and Swedish workers spent more than 10 hours per day being sedentary and most of this time was accumulated in long bouts (cf. Fig. 2). Spending excessive amount of time in long-bout SED corresponds to less variation in physical behaviours [41], which may increase health risks [11, 12]. We observed that, on average, Brazilian workers had more SED in bouts ≥30 min, both during working and non-working days (478 and 359 min) compared to Swedish workers (367 and 251 min). A possible explanation for this finding may be a difference in the homes and workplaces of Brazilian and Swedish workers. As WFH in Brazil was not common before the pandemic, the sudden change in location caused by the pandemic may have caught many of them unprepared. Thus, they had to adapt their homes in the best way possible to carry out their activities. This may have led to workers sitting at their – adapted – workstations for most of the day, not allowing much standing or walking.

Excessive sedentary time, as found in this study, has been associated with detrimental health effects, development of non-communicable diseases and early mortality [9–12]. Until now, there is no exact evidence regarding the time in SED that will lead to a health risk. However, studies indicate that this threshold can be 8 hours or more per day (i.e., about 33% of the day) [10, 12], which is also reflected in the Canadian guidelines [33]. Thus, our results, at least for those workers who spend excessive time in SED (Fig. 2), suggest that strategies should be developed to encourage office workers to be more active. Additionally, interventions implemented in the workplace in the past, such as standing desks for meetings and spatial reallocation of dust bins or printers, may not be useful in the home environment [49]. Therefore, future studies should evaluate the ergonomic conditions when WFH, in order to develop new initiatives to meet recommendations while WFH [47]. In addition, to protect workers’ health, companies should investigate whether the living place fulfil the minimum necessary conditions for work activities to be performed safely.

Specific needs for some workers are suggested by the considerable heterogeneity in the physical behaviour data among workers in both groups (cf. Fig. 2). It appears that some workers may have perceived the pandemic as an opportunity to be more physically active during working and non-working days because work could be structured in a more flexible schedule. In contrast, some may have faced barriers to physical activity introduced by the pandemic, i.e., limited access to public places and closure of sports facilities, thus having fewer possibilities to exercise. This suggests that some workers need additional support, either from their employer or the government through labour and public policies, to deal with conditions when WFH and doing hybrid work.

### Strengths and limitations

A strength of the present study is the use of wearable sensors (i.e., accelerometry) to identify time in different behaviours. By using this type of measurement, we have more detailed and accurate data than if we had used self-reports, and we can evaluate even temporal patterns of SED [25–27, 41]. Our study also showed that the *ActiPASS* program was usable without any problems for data from the devices used in Brazil (ActivPAL Micro4) and in Sweden (Axivity AX3) [36]. Another major strength is the application of a CoDA approach to process the data, which effectively addresses the inherent co-dependency of physical behaviours sharing time within a finite 24-hour window [14, 15].

The Brazilian group consisted only of workers who were recommended to work from home, which may limit the relevance of our results to Brazilian workers who were working in a hybrid model or at the office. Our data did, however, provide evidence about the 24-hour time-use compositions of physical behaviours of a considerable sample of Brazilian office workers WFH during the pandemic. As the prevalence of WFH in Brazil before the pandemic was quite low [22], we believe that our data can help guide future WFH policies. Also, we collected data during different months in Brazil (between September 2020 and April 2021) and Sweden (between June 2020 and June 2021) and this may have influenced behaviours because the pandemic was in different phases. However, we believe this to be a minor criticism. We did not have access to information on the extent to which participants were following public health recommendations at the time of data collection. However, Brazil and Sweden largely relied on recommendations, individual responsibility, and voluntary measures, and likely the fear of contracting COVID-19 reduced people’s motivation to leave their home. Another limitation is that we controlled the analysis only for demographic variables (i.e., sex, age, company position, education and BMI) that were collected in both groups. Additional variables, addressing e.g., socioeconomics, morbidity, health, functional status and household characteristics, could have helped to better understand the differences found between Brazilian and Swedish workers. Notwithstanding these limitations, our study offers a contribution to understanding the physical behaviour of office workers working from home and in a hybrid model during the pandemic in two countries with less severe restriction policies. Thus, our data may help policy makers in creating guidelines for WFH and hybrid work schemes.

## Conclusions

Despite Brazil and Sweden implementing similar restriction policies during the COVID-19, the 24-hour time-use compositions of physical behaviours differed considerably between Brazilian and Swedish office workers. During both working and non-working days, Brazilian office workers were more sedentary and less active than Swedish workers; while time spent in bed was similar among the workers in both countries. Many workers, in particular in Brazil, spent more than half of the 24-hour working day sedentary, predominantly in uninterrupted bouts longer than 30 min. Whether the differences between Brazil and Sweden relate to work tasks, work conditions, or socioeconomic status being different, to restrictions being perceived or interpreted differently, or to differences that were present even before the pandemic is not clear. Given the disease risks associated with extensive sitting, we encourage public health policy-makers to consider our findings when developing future work-from-home guidelines.

## Supplementary Information

**Additional file 1.**

## Data Availability

The datasets generated and/or analysed during the current study are not publicly available due to confidentiality of some data sources, but processed data are available from the corresponding author on reasonable request.

## Abbreviations

COVID-19: Coronavirus Disease 2019
WFH: Work From Home
WAO: Work At the Office
PA: Physical Activity
SED: Sedentary Behaviour
LPA: Light Physical Activity
MVPA: Moderate-to-Vigorous Physical Activity
TIB: Time-In-Bed
non-SED: non-Sedentary Behaviour
CoDA: Compositional Data Analysis
ilr: isometric log-ratio
SD: Standard Deviation
MANOVA: Multivariate Analysis of Variance
MANCOVA: Multivariate Analysis of Covariance
BMI: Body Mass Index
