# Supplementary material for: Sedentary behaviour, physical activity, and sleep among office workers during the COVID-19 pandemic: a comparison of Brazil and Sweden
Source: BMC Public Health. 2022 Nov 28;22:2196. doi: 10.1186/s12889-022-14666-9 (PMC9702952; doi:10.1186/s12889-022-14666-9)
Supplement: Supplementary file 1 — Additional file 1. [file 12889_2022_14666_MOESM1_ESM.docx]

**Additional file 1**

**Supplementary Tables**

Table 1 – Unadjusted and adjusted models including confounders for working and non-working days.

Table 2 – Unadjusted and adjusted models for working days only working from home.

Table 3 – Mean ilr coordinates and results of the univariate post-hoc tests of days working only from home.

**Supplementary Figures**

Figure 1 – Cumulative distributions of percentages of time spent SED in total, SED in bouts <30 min, SED in bouts ≥30 min, LPA, MVPA, and TIB during working days only working from home (WFH).

Figure 2 – Log-ratio with bootstrap 95% percentile confidence intervals of the geometric means of days when Brazilian and Swedish workers worked exclusively from home (WFH).

| **Supplementary Table 1.** Results of the unadjusted models analysing working and non-working days by one-way multivariate analysis of variance (MANOVA); as well as the results of the corresponding adjusted models using one-way multivariate analysis of covariance (MANCOVA) controlling for sex, age, company position, education and body mass index. | | | | |
| --- | --- | --- | --- | --- |
| *Working days* | F (df) | *p*-value | Wilks' *Λ* | Partial eta squared |
| Unadjusted model |  |  |  |  |
| Group | 37.35 (4, 266) | **< 0.001** | 0.64 | 0.36 |
| Adjusted model |  |  |  |  |
| Group | 39.25 (4, 261) | **< 0.001** | 0.62 | 0.38 |
| Sex | 4.40 (4, 261) | **0.002** | 0.94 | 0.06 |
| Age | 2.71 (4, 261) | **0.03** | 0.96 | 0.04 |
| Company position | 1.48 (4, 261) | 0.21 | 0.98 | 0.02 |
| Education | 3.31 (4, 261) | **0.01** | 0.95 | 0.05 |
| Body mass index | 2.02 (4, 261) | 0.08 | 0.97 | 0.03 |
| *Non-working days* | F (df) | *p*-value | Wilks' *Λ* | Partial eta squared |
| Unadjusted model |  |  |  |  |
| Group | 15.32 (4, 249) | **< 0.001** | 0.80 | 0.20 |
| Adjusted model |  |  |  |  |
| Group | 17.40 (4, 244) | **< 0.001** | 0.78 | 0.22 |
| Sex | 3.95 (4, 244) | **0.004** | 0.94 | 0.06 |
| Age | 3.38 (4, 244) | **0.01** | 0.95 | 0.05 |
| Company position | 0.57 (4, 244) | 0.69 | 0.99 | 0.01 |
| Education | 0.76 (4, 244) | 0.55 | 0.99 | 0.01 |
| Body mass index | 5.74 (4, 244) | **< 0.001** | 0.91 | 0.09 |
| Abbreviation: F, F-distribution; df, degree of freedom; *Λ*, Greek letter Lambda. | | | | |

| **Supplementary Table 2.** Results of the unadjusted models analysing days when work was done only from home using one-way multivariate analysis of variance (MANOVA); as well as the results of the corresponding adjusted models using one-way multivariate analysis of covariance (MANCOVA) controlling for sex, age, company position, education and body mass index. | | | | |
| --- | --- | --- | --- | --- |
|  | F (df) | *p*-value | Wilks' *Λ* | Partial eta squared |
| Unadjusted model |  |  |  |  |
| Group | 24.68 (4, 201) | **< 0.001** | 0.67 | 0.33 |
| Adjusted model |  |  |  |  |
| Group | 25.76 (4, 196) | **< 0.001** | 0.66 | 0.34 |
| Sex | 1.82 (4, 196) | 0.13 | 0.96 | 0.04 |
| Age | 2.28 (4, 196) | 0.06 | 0.96 | 0.04 |
| Company position | 3.03 (4, 196) | **0.02** | 0.94 | 0.06 |
| Education | 0.97 (4, 196) | 0.42 | 0.98 | 0.02 |
| Body mass index | 1.43 (4, 196) | 0.20 | 0.97 | 0.03 |
| Abbreviation: F, F-distribution; df, degree of freedom; *Λ*, Greek letter Lambda. | | | | |

| **Supplementary Table 3.** Mean ilr coordinates of the Brazilian and Swedish groups during days worked only from home; as well as the results of the univariate post-hoc tests. | | | | | | |
| --- | --- | --- | --- | --- | --- | --- |
|  | Brazil | Sweden | *t* | MD [95% CI] | *p* | *d* |
| ilr_1_: TIB/wake | 0.92 | 0.77 | 3.86 | 0.15 [0.07; 0.22] | **< 0.001** | 0.59 |
| ilr_2_: SED/non-SED | 1.54 | 0.84 | 8.73 | 0.70 [0.54; 0.86] | **< 0.001** | 1.31 |
| ilr_3_: SEDshort/SEDlong | −0.33 | −0.20 | −1.89 | −0.14 [−0.27; −0.001] | **0.05** | 0.28 |
| ilr_4_: LPA/MVPA | 0.90 | 0.80 | 1.78 | 0.10 [−0.01; 0.22] | 0.07 | 0.26 |
| Abbreviations: ilr, isometric log-ratio; *t*, *t*-test statistic; MD; mean difference from Brazilian group to the Swedish group, 95% CI, lower and upper limit of a 95% confidence interval on the mean difference; *p*, significance level; *d*, Cohen’s effect size *d*; TIB, time-in-bed; SED, sedentary behaviour; LPA, light physical activity; MVPA, moderate-to-vigorous physical activity. Results with p < 0.05 are shown in bold. | | | | | | |


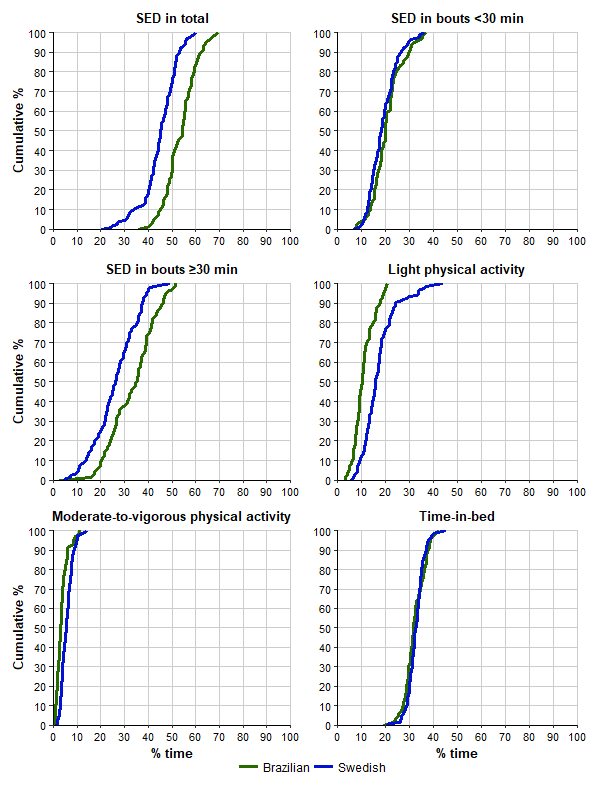


**Supplementary Figure 1.** Cumulative distributions of percentages of time spent sedentary (SED) in total, SED in bouts <30 min, SED in bouts ≥30 min, light physical activity, moderate-to-vigorous physical activity, and in bed. Distributions are shown for Brazilian (green) and Swedish (blue) office workers during work from home only.


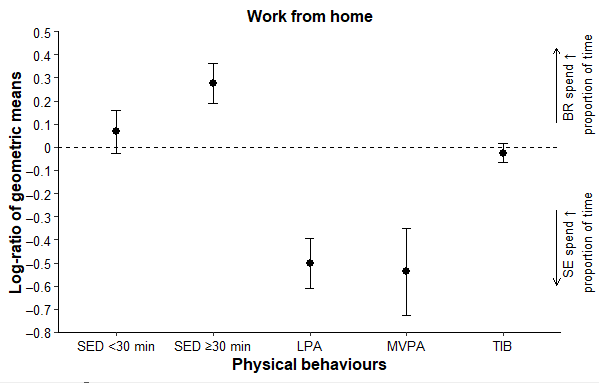


**Supplementary Figure 2.** Log-ratio (circles) with bootstrap 95% percentile confidence intervals (vertical lines) of the geometric means of the Brazilian group (numerator) and Swedish group (denominator) working from home. A positive log-ratio shows that Brazilian office workers spent more time in that behaviour compared to the Swedish office workers, and vice versa if the log-ratio is negative. If the confidence interval includes zero, the difference was not significant at *p*<0.05. Abbreviations: SED, sedentary behaviour; LPA, light physical activity; MVPA, moderate-to-vigorous physical activity; TIB, time-in-bed.
